# Typical Enhancers, Super-Enhancers, and Cancers

**DOI:** 10.3390/cancers14184375

**Published:** 2022-09-08

**Authors:** Marianna A. Koutsi, Marialena Pouliou, Lydia Champezou, Giannis Vatsellas, Angeliki-Ioanna Giannopoulou, Christina Piperi, Marios Agelopoulos

**Affiliations:** 1 Center of Basic Research, Biomedical Research Foundation, Academy of Athens, 4 Soranou Ephessiou St., 11527 Athens, Greece; 2 Department of Biological Chemistry, Medical School, National and Kapodistrian University of Athens, 75 M. Asias Street Bldg 16, 11527 Athens, Greece

**Keywords:** genomic variations, enhancers, Super-enhancers (SEs), transcription factors (TFs), oncogenes, gene expression programs, tumorigenesis, cancer, Next-Generation Sequencing (NGS), chromatin, epigenetics

## Abstract

**Simple Summary:**

The cancer genome has been exhaustively studied upon the advent of Next-Generation Sequencing technologies. Coding and non-coding sequences have been defined as hotspots of genomic variations that affect the naïve gene expression programs established in normal cells, thus working as endogenous drivers of carcinogenesis. In this review, we comprehensively summarize fundamental aspects of gene expression regulation, with emphasis on the impact of sequence and structural variations mapped across non-coding *cis-acting* elements of genes encoding for tumor-related transcription factors. Chromatin architecture, epigenome reprogramming, transcriptional enhancers and Super-enhancers, oncogene regulation, cutting-edge technologies, and pharmacological treatment are substantially highlighted.

**Abstract:**

Non-coding segments of the human genome are enriched in *cis*-regulatory modules that constitute functional elements, such as transcriptional enhancers and Super-enhancers. A hallmark of cancer pathogenesis is the dramatic dysregulation of the “archetype” gene expression profiles of normal human cells. Genomic variations can promote such deficiencies when occurring across enhancers and Super-enhancers, since they affect their mechanistic principles, their functional capacity and specificity, and the epigenomic features of the chromatin microenvironment across which these regulatory elements reside. Here, we comprehensively describe: fundamental mechanisms of gene expression dysregulation in cancers that involve genomic abnormalities within enhancers’ and Super-enhancers’ (SEs) sequences, which alter the expression of oncogenic transcription factors (TFs); cutting-edge technologies applied for the analysis of variation-enriched hotspots of the cancer genome; and pharmacological approaches for the treatment of Super-enhancers’ aberrant function. Finally, we provide an intratumor meta-analysis, which highlights that genomic variations in transcription-factor-driven tumors are accompanied overexpression of genes, a portion of which encodes for additional cancer-related transcription factors.

## 1. Introduction

Regulation of gene expression in metazoans substantially relies on the capability of cells to reconstitute in vivo epigenomic entities endowed with *cis-acting* elements such as transcriptional enhancers and Super-enhancers (SEs), which effectively regulate their target-genes and precisely mount the assembly of gene expression programs. A hallmark of cancer pathogenesis is the dramatic dysregulation of the “archetype” gene expression profiles of normal human cells that characterize their unique identity and specialized functions, which are programmed under homeostasis in a specific tissue microenvironment, where output signals are integrated [1]. The establishment of tumor-oriented aberrant transcriptional states involves the reprogramming of the cellular epigenome, through the means of the structural and functional diversification of its *cis-acting* elements. It is well-addressed that cancer-associated sequence and chromatin architecture alterations are frequently localized within enhancers’ and SEs modules and can have a profound positive impact on tumor-oriented gene expression programs’ establishment [2,3]. Deregulation of transcription factors’ (TFs) function characterizes the pathogenesis of human cancers [4], and consequently the evolution of oncogenic transcription. The impact of a TF in oncogenesis can arise by alterations of coding sequences that lead to structural and functional deficiencies, or abolishment of its synthesis; or by variations across non-coding elements that control its expression, such as nucleotide variants’ incorporation (Single nucleotide polymorphisms (SNPs)), transcription factors binding sites (TFBSs) de novo assembly or disruption, genomic rearrangements or deletions, and *cis-acting* elements’ amplification and hijacking, all of which affect the transcriptional “fitness” of its encoding gene. Such non-coding genomic abnormalities can generate direct or indirect, local or distant aberrant transcriptional regulatory output, which in turn integrates the mechanisms of gene transcription, facilitating the variation of the expression levels. Several TFs (e.g., MYC, MYCN, BCL6, p53) have been exhaustively studied and characterized as master regulators in distinct types of tumors [3,5,6,7,8,9,10], a fact that underscores the current understanding that describes cancer as a deleterious outcome of cellular gene expression dysregulation.

Cancer genomics has been privileged by the application of genome-wide association studies (GWAS) as they enable the investigation of the molecular, genetic, and mechanistic basis of complex malignancies. GWAS have been conducted on a plethora of cancer types, leading to the identification of genetic variants associated with an increased risk of their occurrence and development [11,12]. Striking examples of susceptible sites correspond to variants rs71658797, rs6920364, rs11780471, and rs66759488 (in 1p31.1, 6q27, 8p21.2, and 15q21.1 genomic loci, respectively), associated with lung cancer [13]. Variants rs1550623, rs3903072, and rs2236007 (in 2q31.1, 11q13.1, and 14q13.3 genomic loci, respectively) are risk factors for breast cancer [14], and rs79480871, rs13255292, and rs4733601 (in 2p23.3 and 8q24.21 genomic loci, respectively) increase the risk of DLBCL [15].

Enormous effort has been undertaken in the fine mapping of variations of the cancer genome via the application of Next-Generation Sequencing (NGS) technologies [1], specialized in the analysis of recurrent abnormalities [16] (Table 1 and Table 2). Cancer genomics has benefited from strategies that apply transcriptomics, chromatin accessibility assays, epigenetics profiling, genome-editing, and computational biology approaches, at the cell-population or single-cell level. This powerful repertoire of methodologies can integrate precious findings and deliver mechanistic understanding beyond the levels of the mere description of DNA sequence alterations or/and phenotypic characterization, because it enables the dissection of gene expression programs and cellular epigenome reprogramming, as established in cancers (Table 1 and Table 2). Thus, revolutionary technologies and integrative approaches can lead to the functional elucidation of the molecular mechanisms that direct the non-coding-driven transformations of somatic cells in liquid or solid tumors, and have been successfully applied in blood, bone marrow, lymph nodes, breast, prostate, bladder, lung, skin melanoma, brain, and epithelial cells/tissues systems of study [1,16,17,18]. Numerous effective cancer-related variations in non-coding/regulatory sequences have been shown to impact the epigenome architecture and lead to local, regional, or genome-wide scale reprogramming of enhancers’ and SEs landscapes, with dramatic ramifications for the maintenance of cellular fates. Recent advantages illuminated the significance of enhancers’ and SEs’ (epi)genomic profiling and functional investigation for approaching the cellular mechanisms of cancer development, the identification of clinical stratifications, and the efficient drug design and prediction of dependencies, all of which can have a substantial contribution to therapeutic applications’ development [19,20,21].

Here, we focus on fundamental discoveries that illuminate the impact of non-coding genome alterations in the establishment of aberrant gene expression programs accompanying cancer phenotypes’ evolution. We provide comprehensive descriptions of mechanistic studies that addressed in-depth the interplay between DNA sequence variations, epigenetic characteristics, functional traits, and malfunctions acquired in tumor-related enhancers and SEs of genes that encode for cancer-related TFs, defined in distinct human tissues.

## 2. DNA Grammar and Syntax Features and Epigenetic Characteristics of Enhancers and Super-Enhancers Landscapes

Non-coding segments of the human genome are enriched for *cis*-regulatory modules (CRMs) and host the vast majority of disease-associated genetic polymorphisms [44]. The essential role of regulatory CRMs in human physiology is suggested by the deleterious effects derived from alterations within their composite DNA sequences, which can lead to cellular homeostasis misregulation and development of severe pathogenic phenotypes [44,45] including cancer [46]. These deficiencies primarily arise as outcomes of aberrant gene expression programs establishment. *Cis-acting* elements are composed of modular stretches, are broadly distributed throughout human chromosomes, and reside proximal, distal [47], or entirely coinciding with their target-genes. The human genome was shaped throughout evolution [44], and has acquired an enriched lexicon of transcription factor binding motifs (TFBMs) assembled under the function of the mechanisms of natural selection. The in vivo operation of *cis-acting* elements is founded on their capacities to integrate signals and host TFs that mechanistically interpret the DNA code and coordinate the generation of transcriptional regulatory output, which grants the fine-tuning of gene-transcription in a stimulus-, cell-type-, spatiotemporal-, and quantitative-specific mode. Precise gene expression control is vital for human development and homeostasis’ maintenance and can be dramatically affected when point, local, or regional sequence alterations occur. Therefore, enhancers and SEs commit to the establishment of gene expression programs that characterize the unique identity and specialized functions of individual cell types [18], across the human body segments.

Typical enhancers are short, routinely span 100–1000 bp of DNA sequence [48], and harbor DNA-binding sites (TFBSs) [49], which are recognized, by diverse affinities and specificities, by sequence-specific TFs [44]. Protein–DNA associations direct the interplay between enhancers and TFs, which is based on strong, mild, or weak, reliable TFBMs-DNA-Binding-Domains’ (DBDs) biochemical interrelationships [50,51,52]. The variability of TFBMs reflects/indicates the complexity of the primary DNA sequences of the non-coding segments of the genome and ensures the assembly of a wealth of divergent enhancers composed of alternative modules, diversities, and arrangements of TFBSs that are recognized by a multitude of families and combinations of TFs. On the basis of TFBSs composition, enhancers can encompass a limited number or a series of homotypic or degenerate binding sites for the same TF or diverse TFBSs recognized by distinct TFs [44,53]. Importantly, the order, orientation, spacing, and exact number of TFBSs imprint a unique syntax within each enhancer [52], critical for its in vivo operation. These features are essential for modulating gene activation or repression [54], according to cell-type-, stimulus-, spatiotemporal- and quantitative-specific applied rules. Enhancers work efficiently independent of the chromosomal position (same or different chromosome), linear spacing, and orientation relative to the Transcription Start Site (TSS) of their target-genes [46,55,56]. Transcriptional activators or repressors bind individually or cooperatively to TFBSs within enhancers [46] and recruit via their trans-activation domains regulatory partners, such as cofactors and chromatin remodelers/modifiers. Thus, apart from TFBM–DBD and TF–TF contacts, a wide network of protein–protein associations is developed on enhancers as a matter of TFs’ DNA-binding specificities [57,58], strength [51], and DNA-induced allosteric conformations [35,59]. These affinity-dependent capacities of TFs to assemble multi-protein complexes and recruit cofactors and chromatin remodelers/modifiers are essential for the structure of higher-order transcriptional regulatory complexes [54,60,61] acting on chromatin landscapes. In addition, chromatin states’ accessibility (open vs closed states) [30,62] modifications on histone octamers (e.g., acetylation, methylation, phosphorylation, ubiquitination) [63,64,65,66], histone variants incorporation [35,56,67], and histone–DNA contacts’ remodeling [66] shape the chromatin microenvironment across which an enhancer resides, and substantially program its functional state and the transcriptional status (activation or repression) of its target-gene(s). Overall, chromatin architecture and epigenetic characteristics have a profound impact on CRM-directed gene expression regulation [56] in the context of the assembly of transcriptionally “compatible” or “inhibitory” chromatin microenvironments.

A pervasive characteristic of enhancers is their ability to become self-transcribed and produce non-coding RNAs termed enhancer RNAs (eRNAs) [68,69,70,71,72]. The functional impact of eRNAs on gene expression regulation phenomena is broadly applied in diverse cellular functions and malfunctions, such as cell development [68], differentiation [73], and cancer progression [69,70]. In general, the basic mechanistic principles of enhancers’ operation are followed during eRNAs synthesis, through the means of TFs binding, co-activators’ recruitment, and modification of histones with activation marks (e.g., H3K27ac, H3K4me1) [69,73]. Predominantly, eRNAs synthesis has been shown to proceed via bi-directional transcription of enhancers by RNA pol II [69,72]. Importantly, in certain cases, the levels of eRNAs are correlated to those of mRNAs encoded from genes that reside close to the transcribed enhancers [72,74]. In addition, eRNAs influence gene expression regulation by contributing to epigenome architecture, and TF-dependent gene transcription. Interestingly, eRNAs produced from p53-bound enhancer regions (p53BERs) have been shown to execute a functional role in the p53-dependent transcriptional regulation of p53BER-interacting genes [75]. For instance, in malignancies, eRNAs have been shown to contribute to enhancer–promoter communication, e.g., ARIEL, an eRNA that regulates an oncogenic transcriptional program in leukemia by facilitating the recruitment of the Mediator complex and the assembly of 3D associations between the distal enhancer and promoter of the *ARID5B* gene [74,76]. Hence, the capability of enhancers to generate transcriptional regulatory output is exhibited in vivo, through the synthesis of their own transcripts, which in many cases are involved in fundamental cellular phenomena, including cancer-specific gene regulation.

SEs are large entities, occupy expanded regions of regulatory DNA, and are structured as clusters of proximally spaced enhancers excessively marked by histone modifications and loaded by TFs and co-activators [18,54]. In principle, the anatomical patterns of enhancers’ assembly and the molecular mechanisms of their operation are exhibited on SEs structure and function, on the means of the primary DNA sequence, TF binding, and epigenetic characteristics, yet on a more extended scale. However, SEs rely on prolonged segments of the genome spanning more than 10 kb and occasionally reconstitute epigenomic landscapes of more than 100 kb length of DNA sequence [18,77]. These large (epi)genomic “scaffolds” demonstrate superior architecture and are composed of numerous clusters of closely spaced or overlapped enhancers’ stretches lined up in the linear dimension of the genome [78]. SEs contribute both to cell identity and cellular responses [79,80], and the dysregulation of their function has been connected to human diseases development, including cancer [18,19]. Accordingly, both master and stimulus-specific TFs (e.g., OCT4, NF-κB) have been shown to contribute to SEs assembly and function [81,82]. Epigenetic characteristics such as high levels of H3K27ac modification, robust recruitment of bromodomain and extraterminal (BET) domain-containing protein BRD4 and Mediator Complex [21,82], and occupancy of p300 acetyltransferase were introduced as the hallmarks of SEs assembly [21,81]. Further studies assigned additional characteristics such as excessive RNA polymerase II (RNA pol II) recruitment, open chromatin states, and formation of 3D chromatin condensates [83,84,85]. The latter is described in an advanced model of resolving the structure of the 3D perplexed assemblies of phase-separated condensates organized in a “liquid-droplet” fashion, which incorporates multiple SEs, and increased concentrations of co-activators, TFs, and RNA pol II, situated in specific nuclear sub-compartments. The mechanistic logic followed during SEs operation is of paramount interest, since it is not clear yet if their functional outcome equals the sum of the output derived from its constituent enhancer stretches, or if synergistic rules are applied. Notably, several studies utilizing genetic tools to mutate or entirely delete in vivo sub-domains of SEs have highlighted diverse effects on the reduction in gene expression [5,86,87,88]. Additional studies have shown that a specific sub-domain of the SE preserves predominantly the functional capacity, and its deletion leads both to a dramatic decrease in the SE’s operation and an overall dysregulation of its structure [89]. Collectively, our current understanding postulates that SEs constituents can work independently and provide additive or synergistic outcomes or follow temporal and functional hierarchies. Apparently, SEs function relies on their superior architecture, which is supported by the composite sequences and influenced by the local chromatin-microenvironment of residence.

Currently, SE biology is a fast-developed new era of cancer research and advanced studies have highlighted the function of SEs in distinct types and stages, including tumorigenesis, growth, metastasis, etc. In Osteosarcoma (OS), a type of bone cancer, advanced studies have addressed the critical role of SEs in the disease progression [90]. For instance, overexpression of *MYC* and its widespread binding across SEs landscapes characterize OS, while pharmacological treatment of OS cells with SEs inhibitors suppresses proliferation and enhances apoptosis [91]. In addition, the migration capacity of OS cells is decreased upon SEs “drugging” [91]. Moreover, Ewing Sarcoma, another type of bone cancer, is initiated and progressed by the chimeric oncoprotein EWS-FLI1 [90,92], and has been molecularly and mechanistically approached by a SEs angle. In Multiple Myeloma (MM), a malignancy originated from antibody-secreting plasma cells [90,93], fundamental studies have shown the commitment of SEs to the development of the disease. In addition, it has been shown that SEs regulate genes committed in cancer stemness and metastasis, in human head and neck squamous cell carcinoma (HNSCC) [94]. For instance, disruption of SEs by BET-domain pharmacological inhibition, conducted in in vivo models, leads to the elimination of cancer stem cells (CSCs) and suppression of their tumorigenic and metastatic capacities. At the mechanistic level, SEs inhibition hinders the transcription of stemness and pro-metastatic genes [94]. Another advanced study identified a plethora of SEs assembled in esophageal squamous cell carcinoma (ESCC) and metastatic lymph node cancer (LNC) cells [95]. Based on H3K27ac profiling, SEs signatures were precisely defined in primary and lymph node tumors. Apparently, the capability of SEs to maintain cell identity [96] is also applied in diverse cancer cell types that mount unique gene expression programs, a subset of which exhibit chemoresistance. Hence, these large epigenomic entities can be approached as druggable “substrates” during chemotherapies designed against cancer phenotypes progression.

The human genome contains approximately 20,000 coding genes, a subset of which (~1600) encodes for TFs [50], regulated by their own *cis-acting* elements including enhancers and SEs. TFs massively target the genome and mechanistically interpret the imprinted DNA syntax when bound to *cis-acting* elements, leading to the assembly of transcriptional preinitiation complexes. These capabilities of TFs are of paramount importance for life, since they orchestrate the foundation of gene expression. Thus, even a limited alteration of TFs concentration in living cells can account for remarkable transcriptional reprogramming and phenotypic changes. Several studies have shown that the low expression of certain TFs in normal cells and the high expression in cancer cells is controlled by regulatory elements such as enhancers and SEs [2,18]. Sequence variations, such as translocations, deletions, duplications, insertions, point mutations, and focal amplifications, as well as epigenetic changes, conform to this model of pathogenesis. Below, fundamental discoveries in the gene expression regulation of cancer-related TFs are discussed in the context of enhancers’ and SEs’ genomic variations and functional consequences.

## 3. Structural and Sequence Variations of Enhancers and Super-Enhancers Regulate Oncogenic Expression in Human Cancers

### 3.1. Super-Enhancers’ De Novo Assembly in T-Cell Acute Lymphoblastic Leukemia

**Figure 1:**
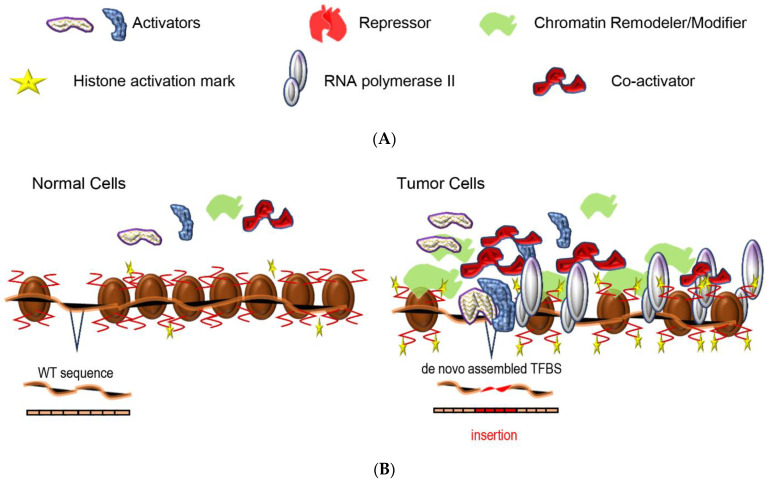

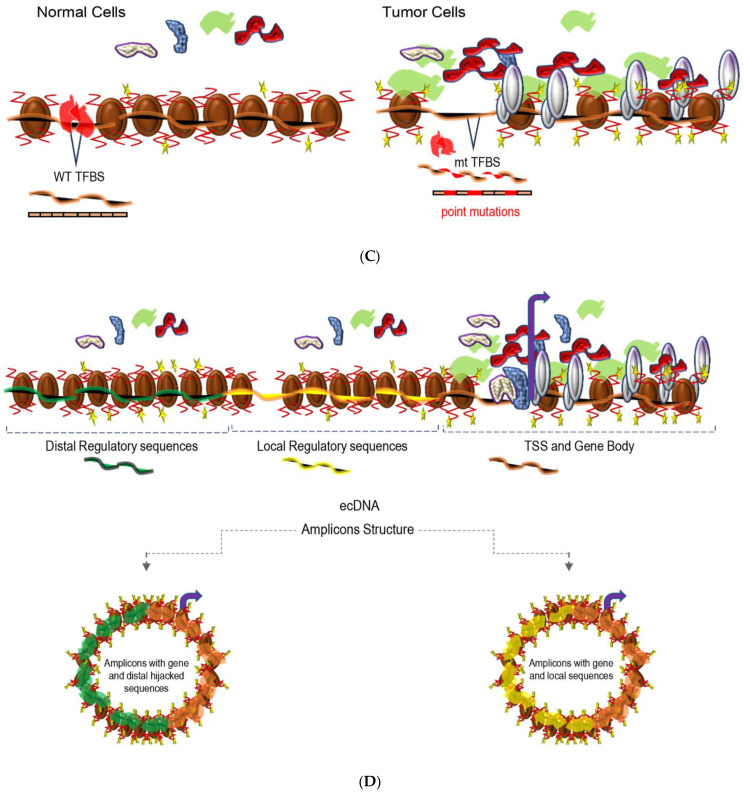
Diagrammatic illustrations of models of enhancers’ and SEs formation in normal and tumor cells: (**A**) Schemes and descriptions of Activators: e.g., MYB, GATA3, RUNX, etc., Repressor: e.g., BLIMP1, NR3C1 (GR), etc., Co-activators: e.g., BRD4, Mediator Complex, etc., Histone activation mark: e.g., H3K27ac, H3K4me1, etc., Chromatin Remodeler/ Modifier: e.g., p300, SWI/SNF, etc.; (**B**) A comprehensive diagrammatic description of SEs assembly via de novo assembly of TFBSs for transcriptional activators, by DNA insertion. Therefore, limited alterations of the primary DNA sequence can have dramatic ramifications for cellular homeostasis by de novo reconstitution of SEs, which can facilitate tumor-oriented transcription. (**A**,**B**); (**C**) A comprehensive diagrammatic description of SEs assembly via the disruption of a binding site for transcriptional repressors, by point mutations. Therefore, the inhibition of transcriptional repressor–TFBS in vivo associations by restricted alterations of the primary DNA sequence when occurring within SEs constituents can promote tumor-oriented transcription; (**D**) A comprehensive diagrammatic description of ecDNA formation. Genes and local or distal regulatory sequences structure diverse types of amplicons in cancer cells. Thus, sequence, structural, and epigenetic changes can occur “episomally” or endogenously and lead to SEs superficial activation and tumor-oriented transcription.

An exceptional paradigm of mechanistic understanding of SE de novo assembly and function derives from studies in T-cell acute lymphoblastic leukemia (T-ALL), a hematological tumor with distinct immunophenotype driven by malignant transformation and expansion of T-cell progenitors [2,97]. The *TAL1* gene encodes for a basic helix-loop-helix (bHLH) TF [98,99], which is aberrantly expressed in T-ALL (>40% of all cases) [100] and its neighboring non-coding loci acquire somatic mutations necessary for the accumulation of TFBMs recognized by the MYB oncogenic TF [2,101]. In particular, the intergenic loci 7.5 kb upstream of the TSS of *TAL1* host excessively increased levels of H3K27ac histone activation mark in Jurkat and MOLT-3 cells but not in other cell types, e.g., normal CD34^+^ hematopoietic stem and progenitor cells (HSPCs), as evidenced by ChIP-seq experiments. This pattern underscored the assembly of a T-ALL-specific SE. Sequencing analysis of genomic DNA identified insertions in the exact locus in cell lines (e.g., Jurkat, MOLT-3) and patient-derived samples. In silico analysis highlighted enrichment of TFBMs recognized by MYB and consequently defined the acquirement of its binding site within the −7.5 kb locus, leading to the assembly of a new/modified *cis-acting* element. Additional ChIP-seq assays uncovered the in vivo binding of MYB to the mutated *TAL1* enhancer accompanied by additional factors, members of the TAL1 complex such as GATA3, RUNX1, etc. Furthermore, analysis of the ChIP-seq signals for RNA pol II and Mediator Complex subunit 1 (MED1), which span more than 20 kb, indicated robust transcriptional activation aroused as a matter of the SE formation. Therefore, limited alterations of the primary DNA sequence can have dramatic ramifications for cellular homeostasis and cancer progression via de novo reconstitution of a SE (Figure 1A,B), which leads to aberrant gene expression. The fact that these genomic variations have been found heterozygous in a subset of T-ALL cases [2] and moreover that this is not the only genomic background or epigenomic mechanism linked to *TAL1* overexpression in T-ALL (e.g., TAL1^d^ 90-kb deletion) [102] illuminates the perplexing nature of pathways that are followed during tumorigenesis. 

### 3.2. Hypermutation of Super-Enhancers in Diffuse Large B Cell Lymphoma (DLBCL)

Recently, an advanced study resolved the pattern of hypermutations both at the whole-genome and regional scales and uncovered key mechanistic principles of carcinogenesis in diffuse large B cell lymphoma (DLBCL), a major non-Hodgkin blood cancer [3]. A *cis-acting* elements ranking employed by the application of the ROSE algorithm [81] on H3K27ac-ChIP-seq datasets coupled to computational assessments of the mutation frequency revealed that genomic loci harboring sequences with active SE characteristics encompass significantly higher levels of somatic mutations’ loads compared to other regions, e.g., randomly selected genomic loci, etc. Accordingly, a clear trend of hypermutation incorporation in SEs in DLBCL was recorded. Computational assignment of SEs to candidate genes, implemented based on genome topology-proximity and gene expression patterns, showed a significant correlation to B-cell-specific TFs and proto-oncogenes, including *BCL6*, *BCL2*, *CXCR4*, *CIITA, PAX5,* etc., a fact that underscores the commitment of hypermutated SEs in cellular pathways and functions relevant to the pathogenesis of the disease. The *BCL6* gene encodes for a TF member of the Broad-Complex, Tramtrack and Bric-a-brac/Pox virus Zinc Finger (BTB/POZ) family [9,103], which operates as a repressor and is required for germinal center (GC) B-cell development [104], while its functional deregulation (e.g., by genetic aberrations) has been linked to DLBCL [3,9]. Genomics analysis captured an intragenic SE (iSE) coinciding with the *BCL6* locus, which spans/masks the first intron of the gene, and mapped a sequence stretch of 20 bp length, significantly hypermutated in an enriched percentage among the primary DLBCL cases and cell lines examined. High-resolution analysis revealed that three nucleotide positions [776, 779 and 780] within the stretch of the iSE were frequently mutated in DLBCL samples oppositely to the low-frequency mutation rates observed in normal memory B cells. Genome-editing-based reverting of the mutant to the wild-type (WT) sequence coupled with survival assays and expression profiling proved that mutations of the iSE deregulate *BCL6* levels and conform to addiction in DLBCL phenotype selection. In vitro and in vivo binding assays (EMSA, immunoprecipitation coupled to mass spectrometry, ChIP-qPCR) validated that the sequence stretch of the iSE is a site of the human genome physiologically targeted by BLIMP1. Therefore, the inhibition of transcriptional repressor–TFBS associations by mutations incorporated in the iSE promotes *BCL6* expression deregulation in DLBCL. 

Utilizing an analogous workflow, Bal et al. expanded these findings and exceptionally dissected mechanistic associations and dependencies between mutational hotspots, SEs, and transcriptional regulators, implicated in cancer phenotypes’ development. For instance, the *NR3C1* gene encodes the transcription regulatory factor (TRF) glucocorticoid receptor (GR), which binds in vivo on GR response elements (GREs) and controls the up- or downregulation of expression of its target-genes [105,106]. NR3C1 (GR) targets TFBSs within SEs of *BCL2* and *CXCR4* loci, and mutations in these regions have pronounced effects both on TF’s binding affinity and gene expression levels [3]. Importantly, additional mutations have been previously mapped close to TSS of *BCL6* [107], and defined to facilitate its in *cis*-dysregulation. The disruption of two adjacent *BCL6* binding sites, residing in the first-non-coding exon, affects the capability of the TF to bind to its own promoter, with dramatic deficiencies aroused against its autoregulatory mechanism. Thus, it becomes apparent that mutations are a pervasive feature of SE-harbored loci of the cancer genome, which when hindering the binding of transcriptional regulators such as repressors, can facilitate oncogenic-SEs function (Figure 1A,C), and subsequently the evolution and maintenance of aberrant blood phenotypes addicted to cancer.

### 3.3. Enhancers and Super-Enhancers Regulate MYC Proto-Oncogene Expression in Liquid and Solid Tumors

The TF MYC is encoded by a proto-oncogene and classified to the basic helix-loop-helix leucine zipper (bHLH-LZ) family, while its overexpression is a hallmark of cancer development and derives, among others, as an outcome of diverse genetic backgrounds (e.g., duplications, or somatic mutations) [89,108] or epigenetic mechanisms [5], which stabilize its in vivo abundance. MYC dimerizes with MAX and targets DNA sequences (including E boxes), while its aberrant expression has pleiotropic effects on human homeostasis depending on the cell type, tissue, and somatic microenvironment affected [109,110]. The *MYC* gene resides within a ~3 Mb gene-poor locus which encompasses multiple domains proximal or distal to its TSS sharing epigenomic and functional characteristics of *cis-acting* elements, such as enhancers and SEs, defined in distinct cancer types [5,7,111]. Here, we focused on fundamental paradigms of understanding the mechanistic significance of enhancers’ and SEs formation on *MYC* transcriptional regulation, derived from advanced in vivo studies in liquid (hematological) and solid tumors. An evolutionary conserved genomic locus residing ~1.7 Mb downstream of the *MYC* gene regulates its expression in normal and leukemic hematopoietic stem cells [5]. The “Blood ENhancer Cluster” also known as BENC [5,54,89] is composed of a group of enhancers, and its in vivo reconstitution and operation were approached through the analysis of ChIP-seq data on histone activation marks (e.g., H3K27ac) in diverse cell types [5], and confirmed by endogenized-lacZ reporter (sensors) assays in stem and progenitor cells, whereas its deletion results in a loss of *MYC* expression. Additional genetics-based investigations showed that BENC generates critical regulatory output for the self-renewal, proliferation, and differentiation of hematopoietic stem and progenitor cells. Importantly, assessments on the impact of deletions of individual enhancer domains suggest that BENC works in vivo by utilizing the combinatorial and additive function of its *cis-acting* constituents. Moreover, proximity assays employed by DNA-FISH uncovered intrachromosomal 3D communication of BENC and *MYC*-promoter in hematopoietic stem and progenitor cells. Chromatin accessibility (ATAC-seq) and genomics analysis of ChIP-seq data revealed open chromatin landscapes and a wealth of TF-binding events including those of GFI1b, RUNX, GATA2, MYB associated with the BENC modules [5]. Therefore, analysis of BENC sub-domains showed adjacent and partially redundant CRMs, a structure that indicates the capability of hosting diverse TFs, which can work in a combinatorial mode. Collectively, this cluster of enhancers follows a precisely controlled operation and is active in leukemic cells, a fact that underscores its role in the regulation of hematopoietic malignancies. 

In solid tumors, long-range regulation of *MYC* transcription has also been described [89]. Focal amplifications of lineage-specific SEs have been mechanistically dissected and addressed the assembly and function of SEs, crucial for MYC overexpression in two types of epithelial cancer, lung adenocarcinoma (LUAD) and endometrial carcinoma (UCEC) [112]. Two distinct focal amplification events in 3′ prime non-coding sequences neighboring the *MYC* locus have been mapped at ~450 kb in LUAD and ~850 kb in UCEC cells [112]. In particular, a ~23 kb internal segment of the focal amplicons in LUAD corresponds to a sub-domain of a SE, termed MYC-LASE (lung adenocarcinoma SE), mapped by H3K27ac-ChIP-seq profiling in A549 cells. Computational analysis of copy number variations and genome rearrangement as well as H3K27ac- and p300-ChIP-seq profiling validated the lung adenocarcinoma-specific in vivo assembly of MYC-LASE. Notably, Chromosome Conformation Capture assays (3C) uncovered 3D associations between MYC-LASE and *MYC* promoter, in a cell-type-specific manner. Chromatin accessibility profiling of epigenomic landscapes defined five enhancers constituting the structure of MYC-LASE. Interestingly, one of these enhancers (e3) demonstrates the highest levels of activation marks and executes efficient reporter gene expression, in vivo. TFBSs motif enrichment analysis coupled with TFBSs deletion, reporter-gene-based assays, and ChIP-seq experiments highlighted the binding of CEBPB and NFE2L2 on the e3 enhancer of MYC-LASE. KRAB-dCas9-based deletion of e3 resulted in the reduction of *MYC* expression levels, accompanied by a decrease in H3K27ac abundance. The latter suggests that the SE landscape abolishes both its superior epigenetic architecture and function. Collectively, these results indicate that focal amplifications leading to copy number gain of SEs can impose structural modifications on the (epi)genomic profile and downstream functional alterations crucial for cancer development, in a lineage-specific manner. 

An exceptional paradigm is derived from mechanistic studies in colorectal cancer (CRC), according to which genetic associations have shown that the SNP rs6983267 variant located at the 8q24 genomic locus, [113,114] is linked to the disease development. This variant is mapped ~340 kb distant from the *MYC* locus. High-resolution microarray analysis highlighted that, except for *MYC* coding sequences, the genomic landscapes between the gene and the variant are transcriptionally restrained. This result suggested that the 8q24 genomic locus could work as an enhancer [114]. Indeed, genomic-loci-specific chromatin examination studies focused on enhancers’ activation marks, e.g., p300, H3K4me1, etc., resolved a profile of enrichment indicative of the in vivo assembly of a functional *cis-acting* element. Next, a 1538 base pairs long DNA sequence flanking the SNP rs6983267 was examined in Luciferase reporter assays and revealed significant enhancer activity. In addition, this variant is incorporated in a TFBS consensus, recognized by TFs belonging to the T-Cell Factor family (TCF) [114]. Genome-wide investigation demonstrated that TCF7L2 targets the risk locus, and mass-spectrometry-based analysis highlighted that the variant-enriched TFBS is associated with high efficiency with the TF. Chromosome Conformation Capture assays (3C) uncovered 3D associations between the risk locus and DNA sequences encompassing the *MYC* promoter, as well as a significant portion of its coding region, in CRC cell lines. Importantly, this pattern of physical interactions was not detected in control fibroblast cell lines [114]. Thus, the biochemical and mechanistic evaluation of a genomic locus, which hosts an SNP, sheds light on the assembly of a functional *cis-acting* element that is bound by a transcriptional effector of the WNT signaling pathway, works as enhancer, and is in physical proximity with the *MYC* locus, in CRC cells.

Renal Cell Carcinoma (RCC) is a common type of kidney cancer and is characterized by metabolic abnormalities [115,116]. Hypoxia-inducible factors (HIFs), which regulate cellular responses at the gene expression level under hypoxic conditions, are known to be involved in RCC tumorigenesis. Moreover, GWAS, epigenetics, and gene expression investigations have shown that *MYC* overexpression is linked to RCC development [117,118,119,120]. In particular, GWAS studies identified a common variant located at 8q24.21 associated with RCC [117]. Variant rs35252396 resides in an intergenic locus, 136 kb upstream of *MYC* and ~14 kb downstream of *PVT1*, which encodes for a long non-coding RNA with oncogenic function [117,121]. Grampp et al. conducted a holistic investigation incorporating among others, chromatin studies, meta-analyses of ENCODE data, chromosome conformation applications, and genome editing approaches and addressed that the genomic locus that hosts the SNP demonstrates enhancer characteristics, is bound by HIF, and associates in vivo with both *MYC* and *PVT1* genes. The susceptibility locus hosts binding sites recognized by the HIF, in vivo. Genomics investigations focused on chromatin accessibility revealed enriched signals for open epigenetic states and robust marking by features of active enhancers (H3K4me1 and H3K27ac). The above results suggested the in vivo assembly of a *cis-acting* element. Further mechanistic investigations, implemented by Capture-C experiments, defined the 3D communication of the element with the promoters of *MYC* and *PVT1*, in 786-O cells. Finally, CRISPR/cas9-based disruption of the HIF-TFBSs resulted in reduced HIF-binding accompanied by a limitation of enhancer activation as evidenced by ChIP-qPCR experiments (RNA pol II, H3K27ac). Collectively, this advanced study highlighted that the risk susceptibility locus works as an enhancer, essential for HIF-mediated transactivation of *MYC* and *PVT1* in RCC.

### 3.4. Hijacking of Enhancers and Super-Enhancers, and ecDNA Formation in Tumors

Amplification of proto-oncogenes leads to overexpression of their encoded products and drives carcinogenesis, such as in pediatric neuroblastoma, a type of tumor originating from neural crest cells of the sympathetic nervous system, in children [6,122]. Advanced mechanistic dissections were derived from studies on *MYCN* overexpression [6,123], in which the in vivo function of the amplified regulatory elements was assessed and the sequence, structural, and epigenetic characteristics were defined. *MYCN*-driving enhancers were called by comparison of H3K27ac profiles between expressing and non-expressing cells of non-MYC-amplified neuroblastoma cells lines (e.g., SKNFI, CLBGA), and the results highlighted, apart from the promoter, five *cis*-elements enriched for the histone activation mark in expressing cells, preserving enhancer potential (e1–e5) [123]. Further ChIP-seq studies revealed that four out of the five putative enhancers are bound from TFs, known members of the core regulatory circuit (CRC) critical for neuroblastoma identity [124], PHOX2B, HAND2, and GATA3 [125]. Copy number variation studies uncovered an asymmetric pattern of amplification, according to which, the CRC-bound e4 enhancer is frequently identified in the majority of *MYCN*-amplified neuroblastomas examined. Additional investigations revealed alternative patterns of *cis-acting* elements’ amplifications followed in a tissue-specific fashion. For instance, in sonic hedgehog-driven medulloblastomas (SHH-MB) a SE located more than 350 kb downstream of *MYCN* is consistently co-amplified [123]. Moreover, it was shown that two distinct classes of amplicons are shaped; the first class consistently co-amplifies a proximal enhancer, while the second lacks key local enhancers and contains distal chromosomal loci. Further chromatin accessibility analysis, active histone marks profiling, and 3D assays uncovered that the e4 enhancer is epigenetically primed and operates in vivo, while associating with the *MYCN* promoter. Moreover, amplicons that lack critical local enhancers “regain” their efficiency to drive high levels of *MYCN* expression through the ectopic hijacking of alternative endogenous regulatory elements. Indeed, in the absence of robust local enhancers, various *MYCN* amplicons are structured by incorporating hijacked elements, such as the CRC-driven enhancer, which resides 1.2 Mb downstream of the gene. Importantly, fluorescence in situ hybridization, de novo assembly, and short-read reconstruction methodologies revealed the formation of extrachromosomal circular DNA (ecDNA) amplicons of *MYCN* in the CHP-212 cell line. Moreover, genome topology assessments and Topologically-Associated-Domains (TADs) mapping [126,127] uncovered that chimeric *MYCN* amplicons can reside in new domains termed “neo-TADs” shaped due to chromatin rearrangements. Specifically, in CHP-212 cells, *MYCN* resides at the intersection of two sub-TADs; the first one corresponds to the WT sequence and the second to a distal part of chromosome 2 fused to the *MYCN* locus [123]. Collectively, even though most of the *MYCN* amplicons are constituted by local enhancers, the hijacking of ectopic distal regulatory elements and incorporation into analogous assemblies that lack local sequences can balance the capacity for high levels of gene expression. Therefore, alternatively originated genomic variations can affect neuroblastoma pathogenesis. 

ecDNA formation has been linked to oncogene amplification [128,129] and is excessively found in neuroblastoma [6], for which a wealth of somatically acquired circular molecules of chromosomal origin have been identified. ecDNA molecules contribute to cancer development, progression, and intratumor heterogeneity, and they are considered drivers of focal amplifications, enabling oncogene amplifications and rapid tumor evolution. Importantly, Whole-Genome Sequencing (WGS), Circle-seq, and de novo assembly approaches can detect diverse types of circular DNA structures that encompass entire sequences or fractions of genes and non-coding segments. Moreover, apart from *MYCN*, additional genes that encode for cancer-related TFs, e.g., *JUN* and *TAL2*, have been mapped circularized in tumors [6]. Gene expression analysis showed that circular DNA formation is not sufficient on its own to promote overexpression of genes, without being coupled to amplification. Importantly, a major spectrum of neuroblastoma genomic rearrangements was mapped to involve ecDNA formation followed by re-integration in the linear endogenous sequences, a fact that highlights a crucial role of these structures in genome remodeling in cancer [6]. Thus, sequence, structural, and epigenetic changes can occur “episomally” or endogenously and lead to SEs superficial activation (Figure 1A,D) and TF-dependent cancer progression.

**Figure 2:**
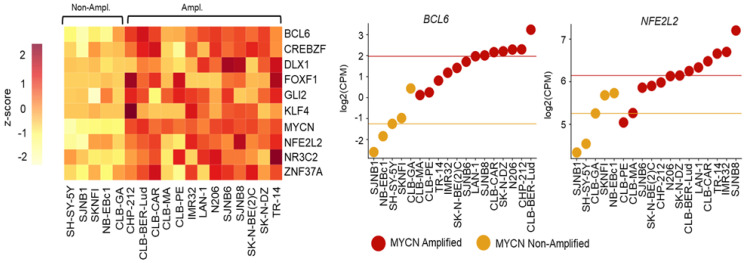
An intratumor meta-analysis of gene expression programs in *MYCN*-amplified and non-amplified neuroblastoma cell lines. A clear trend of upregulation of *MYCN* and additional cancer-related TFs was observed in *MYCN*-amplified samples. (**Left**) A heatmap depicts the expression of each TF-encoding gene examined, across diverse neuroblastoma cell lines (log_2_CPM; Counts per Million). (**Middle**) *BCL6* demonstrates a pattern of elevated expression in *MYCN*-amplified compared to non-amplified neuroblastoma cell lines. (**Right**) same as in Middle for *NFE2L2*.

In this review, we survey a multitude of studies that describe genomic variations in enhancers and SEs that regulate the expression of TF-encoding genes in cancer. We aimed to provide insight relative to our notion that the gene expression programs that are established in cells, which bear such genomic abnormalities, probably are characterized by altered expression of additional TFs apart from the one that its *cis-acting* elements are affected by. We took advantage of published RNA-seq datasets [125] and we conducted a meta-analysis centered on the identification of upregulated TFs in model systems of TF-dysregulated cancers. We retrieved RNA-seq datasets from two distinct classes of neuroblastoma cell lines, which differ in *MYCN* amplification [123,125], and we performed an intratumor transcriptomics analysis. From the voluminous datasets emerged, we fished out the top 506 Differentially Expressed Genes (DEGs) that display |log_2_[fold-change (FC)]| ≥ 1 and *p*-value < 0.01, between the two classes of neuroblastoma cell lines. Next, we further selected from the total DEGs those that captured upregulated in *MYCN*-amplified compared to non-*MYCN*-amplified cell lines. We intersected our results with the official list of human TFs charted in Lambert et al., and we defined approximately 40 genes encoding for TFs, overexpressed. Apart from *MYCN,* genes encoding for well-characterized oncogenic TFs such as *BCL6*, *NFE2L2*, *KLF4*, *GLI2*, etc. [130,131,132,133], follow a consistent profile of enriched expression in cell lines where *MYCN* is amplified (Figure 2, left heatmap). Additional individual analysis of the expression pattern of key oncogenic TFs verified this clear trend of elevated expression among the transcriptomes of the *MYCN*-amplified compared to the non-*MYCN*-amplified neuroblastoma cell lines (Figure 2, center and right, dot plots). Apparently, these results suggest that upregulated DEGs are enriched for oncogenic TFs in *MYCN*-associated tumors, such as neuroblastoma. Thus, further studies should be oriented towards the examination of the assembly of putative regulatory networks of TFs that commit in such cancer-related dependencies. 

## 4. Cutting-Edge Technologies for Mapping of Genomic Variations in Cancer

Genome-wide NGS-based technologies are broadly utilized in oncology for variant detection, gene expression profiling, neoantigen prediction, and liquid biopsies, thus largely contributing to diagnosis, prognosis, and therapy selection [134] (Table 2). 

**Table 2:** Cutting-edge technologies utilized for the analysis of the cancer genome.

Technology	Description	Reference No.
WGS: Whole-Genome Sequencing	Whole-Genome Sequencing (WGS) is utilized to detect mutational signatures related to structural and nucleotide variants in both coding and non-coding regions of the entire genome, including those located at loci where enhancers and Super-enhancers (SEs) are assembled.	[135,136]
TS: Targeted DNA Sequencing	Targeted DNA Sequencing, ranging from Whole-Exome Sequencing (WES) to small custom Gene Panels, allows high-resolution analysis (depth and coverage), thus enabling the detection of low-frequency somatic variants in targeted genomic loci. Following the increasing numbers of cancer-driver gene-mutations currently identified, targeted gene panel sequencing is an efficient application for diagnosis and/or prognosis in clinical laboratories.	[137,138]
OGM: Optical Genome Mapping	The novel emerging method of Optical Genome Mapping (OGM) demonstrates a powerful tool for efficient detection of structural variants, such as copy number variations (CNV), translocations, inversions, and focal amplifications in expanded regulatory elements, such as SEs. The tool is based on fluorescent-labeling of specific sequence motifs and de novo genome assembly, which is then compared to the reference genome. This approach bridges the gap between WGS and karyotyping. OGM de novo genome assembly allows the construction of extended maps (e.g., of a whole chromosome arm).	[139]
Circle-seq: Circular DNA Sequencing	Sequence identification of extrachromosomal DNA (ecDNA) is feasible through Circle-seq. This is an NGS method that relies on the isolation of circular DNA through enzymatic degradation of linear DNA in the sample, efficient amplification of ecDNA, library construction, and sequencing coupled to bioinformatics analysis with custom mapping software, thus achieving identification at the single amplicon level. Focal amplifications, a recurrent signature in a variety of human cancers, involve the formation of a single amplified region of several rearranged DNA fragments from distinct chromosomal loci and Circle-seq is an effective approach to exploring focal amplifications in clinical samples.	[6,140,141]
Long-Read Sequencing	The recent development of the 3rd generation long-read technologies (e.g., PacBio single molecule real-time (SMRT) technology and Oxford Nanopore Technology) has provided several advantages in cancer research. SMRT sequencing can obtain reads longer than 10 kb, whereas nanopore sequencing can provide reads up to 32 kb or even ultra-long reads up to 800 kb. Those capabilities enable extremely efficient characterization of cancer-associated structural variants (SVs), such as large insertions, deletions, inversions, duplications, and translocations eliminating in silico molecule reconstruction uncertainties. Furthermore, Oxford Nanopore long-read real-time sequencing allows the direct identification of epigenetic marks in native DNA and RNA.	[142,143,144,145]
Liquid Biopsy	Liquid biopsy NGS is lately emerging as a powerful tool in cancer detection. WGS, targeted panel Seq, or RNA-Seq can be performed with Circulating Tumor Cells (CTCs), tumor cell-free DNA (cfDNA), extracellular vesicles (EVs), and non-coding RNAs (ncRNAs) that are detected in various bodily fluids (blood, cerebral spinal fluid, urine, and others). This methodology can provide diagnosis, early detection of tumors, and identification of novel biomarkers.	[146,147,148]

## 5. Pharmacological Treatment and Super-Enhancers in Cancers

Several studies revealed that enhancers and SEs could be targeted pharmacologically in cancer therapies [149,150]. BRD4 and MED1 are two crucial transcriptional co-activators, which are key components of SEs and participate in the generation of phase-separated condensates in regulatory regions controlled by SEs [18,81,83,151]. Furthermore, both co-activators have been shown to be enriched and co-localize in SEs, a pattern that suggests a synergistic mode of operation. An effective small-molecule inhibitor of SEs is the BET inhibitor, which targets the BRD co-activators (BRD2, BRD3, BRD4, and BRDT). In particular, BRD4 recognizes acetylated histones, interacts with MED1, and participates in the regulation of transcriptional elongation through associations with RNA poI II [152]. JQ1 is a BET inhibitor studied in myeloma cells, which blocks BRD4 oncoprotein binding to SEs and selectively hinders transcription of *MYC* [21]. Furthermore, in acute myeloid leukemia (AML) cells, the JQ1 inhibitor has been shown to cause the release of MED1 from regulatory regions, which is associated with transcriptional repression [4]. Thus, JQ1 reduces the occupancy of BRD4, the recruitment of MED1, and slows down the elongation of RNA pol II [4,21,152,153]. In addition, BRD4-targeting BET inhibitors such as BI-89499, OTX015, and CPI0610 [21,77,154,155,156] can be effectively applied against liquid and solid cancer types (e.g., neuroblastoma, medulloblastoma, acute myeloid leukemia) [157,158,159].

Another group of druggable molecules associated with SE-dependent transcription is the cyclin-dependent kinases (CDKs; CDK7 and CDK9), which appear to be considerable targets for cancer therapy. CDKs are members of the serine/threonine kinase family, are involved in the cell cycle and RNA pol II phosphorylation-mediated transcription [160], and affect the regulation of SE-driven oncogenes [161]. For this reason, CDKs are considered targets for the treatment of various cancers, as their inhibitors can reduce the levels of oncogenic TFs [162]. THZ1 targets CDK7, and causes the dysregulation of TFs regulated by SEs, such as RUNX1 in T-ALL cells [163] and MYCN in neuroblastoma cells [164]. 

Additional targets are chromatin regulators, which are found dysregulated in cancers [165]. AU-15330 is an anticancer reagent, which aims to limit the function of SEs, through the degradation of the SMARCA2 and SMARCA4 subunits of the chromatin regulator, SWI/SNF [166]. The effect of AU-15330, which is a proteolysis-targeting chimera (PROTAC) degrader, on prostate cancer cells resulted in the destruction of SWI/SNF thereby disrupting SE-promoter communication of *AR*, *FOXA1*, and *MYC* oncogenes, reducing their expression [166]. In addition, a recent study in glioblastoma (GBM) models showed that histone deacetylases (HDACs) such as HDAC1 and HDAC2, which remove acetyl groups from histones, are druggable [167]. More specifically, HDAC inhibitors, panobinostat (Pb) and romidepsin (Ro), cause a reduction in the levels of the c-MYC through the disruption of the SE of the *MYC* gene, consequently reducing the glycolysis of cancer cells [167]. 

Accordingly, it has been well-addressed that enhancers and SEs are druggable and sensitive to pharmacological inhibition of transcriptional co-activators and chromatin regulators. Therefore, these pharmacological approaches sound promising for cancer treatment.

## 6. Integrated Databases of Enhancers and Super-Enhancers 

A variety of enhancer and SE databases have been developed and provide comprehensive resources, enabling the functional annotation of the human genome. FANTOM5, the fifth iteration of the FANTOM project, contains a wealth of actively transcribed regulatory elements (promoters and enhancers) of the human genome captured in high resolution by the application of CAGE (Cap Analysis of Gene Expression) [29]. The FANTOM5 repository includes enhancers and SEs monitored in approximately 1800 human and approximately 1000 mouse samples derived from organs and tissues, including primary cell types and cancer cell lines. In FANTOM5, *cis*-regulatory element profiling expands beyond the naïve cellular states and includes regulatory elements identified in cells during consecutive stages of differentiation and response to diverse stimuli [168,169]. HACER is an atlas of human active and in vivo transcribed enhancers, which annotates nearly 1,600,000 elements in approximately 260 human cell lines through the integration of the FANTOM5 CAGE profiles and reanalysis of publicly available nascent RNA sequencing experiments utilizing GRO-seq and PRO-seq, ENCODE ChIP-seq data, and data validated from chromosome conformation capture-based (3C) technologies [170]. EnhancerDB is a web-accessible database containing data of 41 human tissue or cell lines. EnhancerDB is not limited to mere functional annotation of enhancers but extends to expression quantitative trait locus (eQTLs) SNPs, gene expression data, and transcription factor binding data through the analysis of ENCODE ChIP-seq experiments [171]. dbSUPER is an integrated SE database [172] including both previously published SEs from human and mouse cell lines and additional SEs derived from publicly available H3K27ac-, MED1-, and BRD4-ChIP-seq datasets processed with the ROSE algorithm. Moreover, each SE in dbSUPER is associated with a transcribed gene based on its proximity in cases where experimentally identified SE-gene associations are not available [172]. SEdb is a database that documents SEs from approximately 540 samples and combines information for common and risk SNPs, eQTLs, TFBS, CRISPR/Cas9, and DNase I hypersensitivity sites [173]. The SEs were identified utilizing the ROSE algorithm with publicly available H3K27ac-ChIP-seq data from NCBI/GEO, ENCODE, and the Roadmap Epigenomics Project [173]. CancerEnD is a digital resource developed to provide information regarding enhancers that are associated with tumorigenesis [174]. The database annotates, among others, approximately 8500 unique expressed enhancers, target-genes, somatic mutations, and copy number variations (CNV) [174].

## 7. Conclusions and Future Directions

This review summarized fundamental discoveries that force our current understanding of gene expression regulation in cancers several steps ahead. The capability of TFs to transform the state of a cell is a hallmark of cancer research. Lessons from HOX genes (homeodomain-containing) in *Drosophila* illuminated our understanding relative to the homeotic transformations of cells, tissues, and organs [175]. Studies in cellular reprograming of somatic cells into induced pluripotent stem cells (iPSCs) underlined the capability of TFs to convert cellular fates by modifying gene expression programs [176]. Cancer has been approached as a disease of the genome, the epigenome, and gene-expression regulation. TFs are critical molecular components that function in such abnormalities of living cells, which mechanistically interpret the DNA code, modify the epigenome landscapes, and generate effective transcriptional regulatory output applied during gene expression regulation. Thus, the balance between TFs concentrations, enhancers and SEs sequence and structural integrity, and cellular homeostasis is of paramount importance for life. We envision that TF-oriented research on enhancers and SEs should be broadly conducted on model systems and clinical specimens, by utilizing cutting-edge sequencing-based technologies, in order for such complex phenomena of cancer pathogenesis to become entirely resolved.

## 8. Methodology: Bioinformatics Analysis

RNA-sequencing FASTQ files of human neuroblastoma cell lines were downloaded from the Gene Expression Omnibus (GEO; https://www.ncbi.nlm.nih.gov/geo/, accessed on 3 August 2022) database (GEO accession number GSE90683) [125]. We meta-analyzed publicly available data retrieved from two groups of neuroblastoma cell lines classified according to *MYCN* amplification. The first group without *MYCN* amplification was composed of 5 neuroblastoma cell lines, while the second group with *MYCN* amplification contained 13 neuroblastoma cell lines. Information about samples can be found in ref. [125]. RNA-sequencing data were analyzed by the application of the Galaxy online data analysis platform [177]. The quality of sequencing reads was evaluated by the application of FastQC (Galaxy Version 0.73+galaxy0). Next, reads were aligned on the hg19 reference human genome, using the HISAT2 alignment tool (Galaxy Version 2.2.1+galaxy0) [178] with “paired-end data from single interleaved dataset”, “stranded”, and “reverse” options. The calculation of sequencing reads that are mapped across the genes was performed by the application of the htseq-count tool (Galaxy Version 0.9.1+galaxy1) [179], in union mode, with “stranded”, “reverse,” and “minimum alignment quality 10” options. Differentially Expressed Genes (DEGs) were identified by utilizing the edgeR algorithm (Galaxy Version 3.36.0+galaxy0) [180], coupled with filtering out genes with less than 10 counts in at least 5 individual samples. Statistically significant DEGs were identified by applying *p*-value threshold < 0.01 and |log_2_[fold-change (FC)]| ≥ 1. The datasets of genes that emerged were screened for TFs by intersection with the official list of human TFs [50]. 

## Figures and Tables

**Table 1:** A toolbox of advanced methodologies for enhancers’ and Super-enhancers’ identification.

Methodology and Applications	Basic Principles	Reference No.
RNA-seq: RNA-Sequencing Gene Expression Programs Analysis	RNA-seq harvests intact cells, tissues, or organs. Total isolated RNA is subjected to poly(A)-based mRNAs selection, followed by reverse transcription, cDNA synthesis, and multiplexed libraries construction, which are analyzed by Next-Generation Sequencing (NGS) and bioinformatics applications.	[22,23]
GRO-seq: Global Run-On Sequencing Nascent RNA Profiling and Assessment of the Role of Non-coding RNAs	GRO-seq is applied on isolated nuclei and is based on a nuclear run-on (NRO) reaction, a method, which ensures that RNA pol II and other proteins remain associated with genomic sequences. The activation of transcription and the incorporation of labeled ribonucleotides are followed by sequential steps of isolation, fragmentation, and precipitation of NRO-RNAs and construction of NRO-cDNA multiplexed libraries, which are analyzed by NGS and bioinformatics applications.	[24,25,26]
PRO-seq: Precision Nuclear Run-On Sequencing Genome-Wide Distribution of RNA pol II at a Single Base-Pair Resolution	PRO-seq is utilized for precise mapping of RNA pol II localization with base pair resolution, without the need for an immunoprecipitation step. Cells are harvested, nuclei are isolated, and NRO reactions drive the RNA-pol-II-dependent incorporation of labeled nucleotides into the 3′ end of nascent RNAs. The labeled RNAs are subjected to multiplexed libraries’ preparation and are sequenced from the 3′ prime end, a step that ensures the precise mapping of the active site of RNA pol II associated with nascent RNAs. NGS and bioinformatics applications are utilized for the analysis of the results.	[27,28]
CAGE: Cap Analysis Gene Expression In Parallel Assessment of Eukaryotic Capped RNAs and Mapping of Promoter Regions	CAGE is specialized in detecting the RNA expression levels, mapping the Transcription Start Sites (TSS) in promoters, and identifying the exact promoter that controls the synthesis of each transcript generated, in vivo. The method is based on the extraction of total mRNA, reverse transcription using random primers with EcoP15I site, biotinylation of the RNA cap and 3′ ends, digestion of non-hybridized single-stranded RNAs with RNaseI, and capture of 5′ complete cDNAs on streptavidin magnetic beads. The cDNA is released from the RNA and subjected to the construction of multiplexed libraries, which are analyzed by NGS and bioinformatics applications.	[29]
DNaseI-seq: DNaseI-Sequencing Chromatin Landscapes Accessibility Profiling	DNaseI chromatin accessibility assay is directly applied to intact isolated nuclei without the need for a previous fixation step and is based on the ability of the enzyme to partially digest chromatin filaments when limited concentrations are utilized. The digested chromatin filaments are subjected to size selection and multiplexed libraries’ construction, which are analyzed by NGS and bioinformatics applications. The results obtained are indicative of discriminating between the in vivo topographies of “open” or “closed” chromatin states and mapping of putative enhancers and Super-enhancers (SEs) that preserve high activation potential, in vivo.	[30,31]
ATAC-seq: Assay for Transposase-Accessible Chromatin Chromatin Landscapes Accessibility Profiling	ATAC-seq maps chromatin accessibility, TFs binding, and identifies enhancers, and is based on the function of a mutated hyperactive Tn5 transposase. Tn5 Transposase fragmentizes chromatin filaments and adds sequencing adapters into genomic DNA fragments derived from open chromatin sites, a process called “tagmentation”. The fragments of DNA are purified, PCR-amplified, and subjected to multiplexed libraries’ construction, which are analyzed by NGS and bioinformatics applications.	[32,33,34]
ChIP-seq: Chromatin Immunoprecipitation Sequencing Epigenetics Profiling of Enhancer Activation Marks	ChIP-seq is a method primarily applied to fixed cells, ideal for the identification of the on-genome distribution of transcriptional regulators and epigenetic characteristics. The isolated chromatin filaments are subjected to enzymatic- or ultrasonic-based shearing followed by probing with antibodies against enhancers’ and SEs’ features such as transcription factors (TFs), Co-activators, and Histone activation marks (e.g., H3K27ac, H3K4me1). The ChIPed DNA fragments are subjected to multiplexed libraries’ construction, which are analyzed by NGS and bioinformatics applications.	[35,36]
STARR-seq: Self-Transcribing Active Regulatory Region Sequencing Massive-in-Parallel in vivo Functional Examination of *cis*-Regulatory Elements	STARR-seq protocol is based on the ability of regulatory elements to activate transcription from a distance, even when they reside several kbs downstream of a promoter element. The regulatory sequences of examination are massively recombined in STARR-vectors immediately downstream of the translational stop codon of a reporter gene and intermediately upstream of the poly(A) signal, generating episomal plasmid libraries, transfected in the nucleus. A functional *cis-acting* element can activate its own transcription from a distance and become self-transcribed, as a chimera with the sequence of the reporter gene, in vivo. The STARR-transcripts are subjected to reverse transcription and cDNA synthesis followed by multiplexed libraries’ construction, which are analyzed by NGS and bioinformatics applications. The in vivo levels of enhancers’ activation are evaluated based on the number of NGS-reads obtained.	[37,38]
CRISPR/Cas9: Clustered Regularly Interspaced Short Palindromic Repeats/CRISPR-Associated Protein 9	CRISPR/Cas9 is a revolutionary genetic engineering technique that can modify with precision the DNA of living organisms at will. Genome editing with the CRISPR/Cas9 method relies on the type II CRISPR system. High-throughput CRISPR/Cas9 technology can be utilized in studies focused on the treatment of genetic and hereditary diseases.	[39,40,41,42,43]

## Data Availability

RNA-sequencing FASTQ files of human neuroblastoma cell lines were downloaded from the Gene Expression Omnibus (GEO; https://www.ncbi.nlm.nih.gov/geo/, accessed on 3 August 2022) database, GEO accession number GSE90683, and information about samples can be found in Boeva, V et al., 2017 [125].
